# DGCLCMI: a deep graph collaboration learning method to predict circRNA-miRNA interactions

**DOI:** 10.1186/s12915-025-02197-9

**Published:** 2025-04-23

**Authors:** Chao Cao, Mengli Li, Chunyu Wang, Lei Xu, Quan Zou, Yansu Wang, Wu Han

**Affiliations:** 1 Institute of Fundamental and Frontier Sciences, University of Electronic Science and Technology of China, Chengdu, Sichuan 611731 China; 2 Yangtze Delta Region Institute (Quzhou), University of Electronic Science and Technology of China, Quzhou, Zhejiang 324003 China; 3 Faculty of Computing, Harbin Institute of Technology, Harbin, Heilongjiang 150001 China; 4 School of Electronic and Communication Engineering, Shenzhen Polytechnic University, Shenzhen, Guangdong 518055 China; 5 Department of Statistics, Stanford University, Stanford, CA 94043 USA

**Keywords:** CircRNA-miRNA interaction, Graph neural networks, Collaborative filtering, Word2vec, LSTM

## Abstract

**Background:**

Numerous studies have shown that circRNA can act as a miRNA sponge, competitively binding to miRNAs, thereby regulating gene expression and disease progression. Due to the high cost and time-consuming nature of traditional wet lab experiments, analyzing circRNA-miRNA associations is often inefficient and labor-intensive. Although some computational models have been developed to identify these associations, they fail to capture the deep collaborative features between circRNA and miRNA interactions and do not guide the training of feature extraction networks based on these high-order relationships, leading to poor prediction performance.

**Results:**

To address these issues, we innovatively propose a novel deep graph collaboration learning method for circRNA-miRNA interaction, called DGCLCMI. First, it uses word2vec to encode sequences into word embeddings. Next, we present a joint model that combines an improved neural graph collaborative filtering method with a feature extraction network for optimization. Deep interaction information is embedded as informative features within the sequence representations for prediction. Comprehensive experiments on three well-established datasets across seven metrics demonstrate that our algorithm significantly outperforms previous models, achieving an average AUC of 0.960. In addition, a case study reveals that 18 out of 20 predicted unknown CMI data points are accurate.

**Conclusions:**

The DGCLCMI improves circRNA and miRNA feature representation by capturing deep collaborative information, achieving superior performance compared to prior methods. It facilitates the discovery of unknown associations and sheds light on their roles in physiological processes.

## Background

Because of its unique closed-loop structure, circRNA has a high degree of stability in vivo and is resistant to degradation by RNA enzymes [1, 2]. However, as a special non-coding RNA, due to technological limitations, its important physiological functions were not recognized in the early stages of discovery, and it was generally regarded as a by-product of RNA processing and the result of abnormal gene splicing, which did not attract widespread attention. With the development of high-throughput sequencing technologies in recent years, the physiological function of circRNA has been gradually uncovered [3–5], and many computational models have been proposed accordingly, such as circRNA identification [6–8], circRNA and protein interactions [9–14], circRNA and disease association prediction [15–20], circRNA and drug discovery [21–26]. Among them, one of the most typical areas is the interaction between circRNA and miRNA, which plays a critical role in gene expression, cellular function regulation, and the pathological processes of diseases [13, 27–32]. For example, in tumorigenesis and development [33], circRNA ciRS-7 removes the inhibition of miR-7 on its target genes by adsorption of miR-7, thus promoting the proliferation and migration of tumor cells. In rheumatoid arthritis [34], circRNA hsa_circ_0005198 regulates miR-145, affecting the proliferation and migration of fibroblast-like synovial cells. These studies fully indicate that analyzing the regulatory mechanism mediated by circRNA-miRNA can help people understand the causes of diseases and carry out targeted prevention and treatment. Therefore, the analysis of circRNA-miRNA interactions has great research significance and potential clinical application value.

However, traditional wet lab experiments for validating circRNA-miRNA associations often require long periods for sample preparation and experimental analysis, along with expensive reagents, instruments, and equipment. In addition, they place high demands on the technical level and expertise of researchers. As a result, traditional experiments are often low-throughput, making large-scale, comprehensive, and systematic analyses difficult, which limits further research on circRNA. With the rapid development of big data analysis technologies, many association prediction algorithms have emerged [35–42], which can analyze and model existing interaction data to predict potential unknown associations with high confidence. In subsequent experimental validation, this approach effectively narrows down the most likely candidate objects, reducing the cost and trial-and-error time for experimental verification. The current CMI prediction algorithms are summarized in chronological order below.

In 2021, Lan et al. [43] introduced Gaussian interaction profile (GIP) kernels to calculate the similarity of circRNA and miRNA respectively, and construct a heterogeneous network by associating them with known CMIs. Then, they applied the DeepWalk algorithm [44] based on matrix factorization to extract the latent features of circRNA and miRNA in the heterogeneous network, ultimately predicting unknown associations via regularization, neighborhood information-based logical matrix factorization, and inner-product inference. This algorithm is named NECMA. Qian et al. [45] employed singular value decomposition (SVD) to capture the linear features of the corresponding circRNA and miRNA molecules in CMI and then calculated the multi-similarity of these molecules using Levenshtein distance and GIP kernels. Nonlinear features were extracted using the Variational Graph Autoencoder (VGAE) [46], and LightGBM was used to predict based on both linear and nonlinear features. This algorithm is named CMIVGSD.

In 2022, Guo et al. [47] constructed a new prediction model, WSCD, which used the continuous bag of words (CBOW) model and structural deep network embedding (SDNE) [48] respectively to train word embedding representations as molecular attribute features and graph low-dimensional embeddings as behavioral features. The fused features were then inputted into convolutional neural networks (CNN) and deep neural network (DNN) for circRNA-miRNA association prediction. He et al. [49] proposed a latent interaction prediction model based on graph convolutional networks (GCN) [50], named GCNCMI. Through graph convolution operations, this algorithm effectively mines and propagates complex relationships between nodes, providing deep information for predictions. Yu et al. [51] devised a universal prediction architecture SGCNCMI, which combines multimodal information. Specifically, they first construct fused features using *k*-mer representation to capture RNA’s attribute features and introduce GIP kernels and sigmoid kernels to capture circRNA-miRNA similarity features. Then, the Sparse Autoencoder (SAE) is used to further extract deeper information and serve as feature representations for the corresponding nodes. Finally, a GCN model aggregates adjacent information based on node attributes and association networks to predict potential CMIs. Wang et al. [52] developed an improved algorithm, KGDCMI. They input RNA attribute information, obtained from sequences and similarities via k-mer and GIP kernels, into SAE for representative feature extraction, and use HOPE graph embeddings to mine behavioral information in CMI associations. Finally, a DNN model is applied to fuse features and predict unknown CMIs.

In 2023, Wang et al. [53] introduced a denoising multi-view feature fusion prediction algorithm called JSNDCMI. In detail, it calculates the Jaccard distance between sequences as structural features and uses sigmoid kernels to compute similarities as sequence attribute features. Struc2vec [54] is applied to extract local topological structures from the association network. The multi-view features are then trained with denoising autoencoders (DAE) to obtain more robust feature representations, followed by prediction using GBDT [55]. Zhou et al. [56] designed the SPBCMI model, which combines structural features captured by graph embedding and sequence features extracted by BERT [57]. These features are input into a GBDT classifier for training to complete the CMI interaction prediction task. Wang et al. [58] proposed the KS-CMI algorithm, which constructs a circRNA-miRNA-cancer interaction network based on balance theory to extract molecular behavioral features. Subsequently, DAE is employed to enhance feature robustness, and the CatBoost classifier is used for prediction. Li et al. [59] introduced the DeepCMI model, which integrates molecular similarity matrices and topological information from GIP kernels in biomedical graphs. Multi-source information features are obtained and mapped into the same vector space using local linear embedding, and topological information features are further extracted using text-associated DeepWalk. Finally, an XGBoost [60] classifier is employed to judge whether circRNAs and miRNAs interact. Wei et al. [61] proposed the BCMCMI model, which combines semantic features of sequences captured by BERT networks, features from cosine similarity, and topological features of heterogeneous networks captured by Metapath2vec [62], training an XGBoost classifier to predict potential CMIs.

In 2024, Guo et al. [63] put forward a new prediction algorithm, CA-CMA, combining text embedding representation and convolutional autoencoders. Firstly, Skip-Gram is used to obtain RNA embedding features, which are further refined using Convolutional Autoencoders (CAE). Meanwhile, Doc2Vec is employed to capture the semantic features of the sequences. Finally, CMI prediction is performed based on feature fusion using a DNN. Soon after, Guo et al. [64] proposed an improved algorithm BGF-CMAP, which utilized GBDT and graph embedding methods. RNA word embeddings were obtained via Word2Vec [65], and CMI topological features were extracted using graph factorization (GF) and large-scale information network embedding (LINE). These features were fused and input into GBDT for CMI classification.

Although existing CMI prediction models have achieved relatively good prediction performance through various feature embedding algorithms and efficient neural network architectures, they generally suffer from the following issues that require further improvement: First, these models overlook the exploration of deep collaborative features in circRNA and miRNA interactions. Secondly, they fail to guide the training of the underlying feature extraction network based on deep collaborative information, making it difficult to obtain representative feature embeddings, which affects the algorithm’s performance.

To address these issues, we introduce and extend the neural collaborative filtering algorithm from the field of recommender systems to circRNA-miRNA association prediction. Specifically, we innovatively construct a neural graph collaborative filtering model (NGCF) combined with a joint training framework for the underlying feature extraction network. An optimized loss function is designed to explicitly guide the training direction of the underlying feature extraction network based on deep collaborative information from circRNA-miRNA interactions. These features are then stored in their respective embeddings as representative features, and the association prediction score can be obtained by calculating the inner product of the corresponding vectors.

**Fig. 1:**
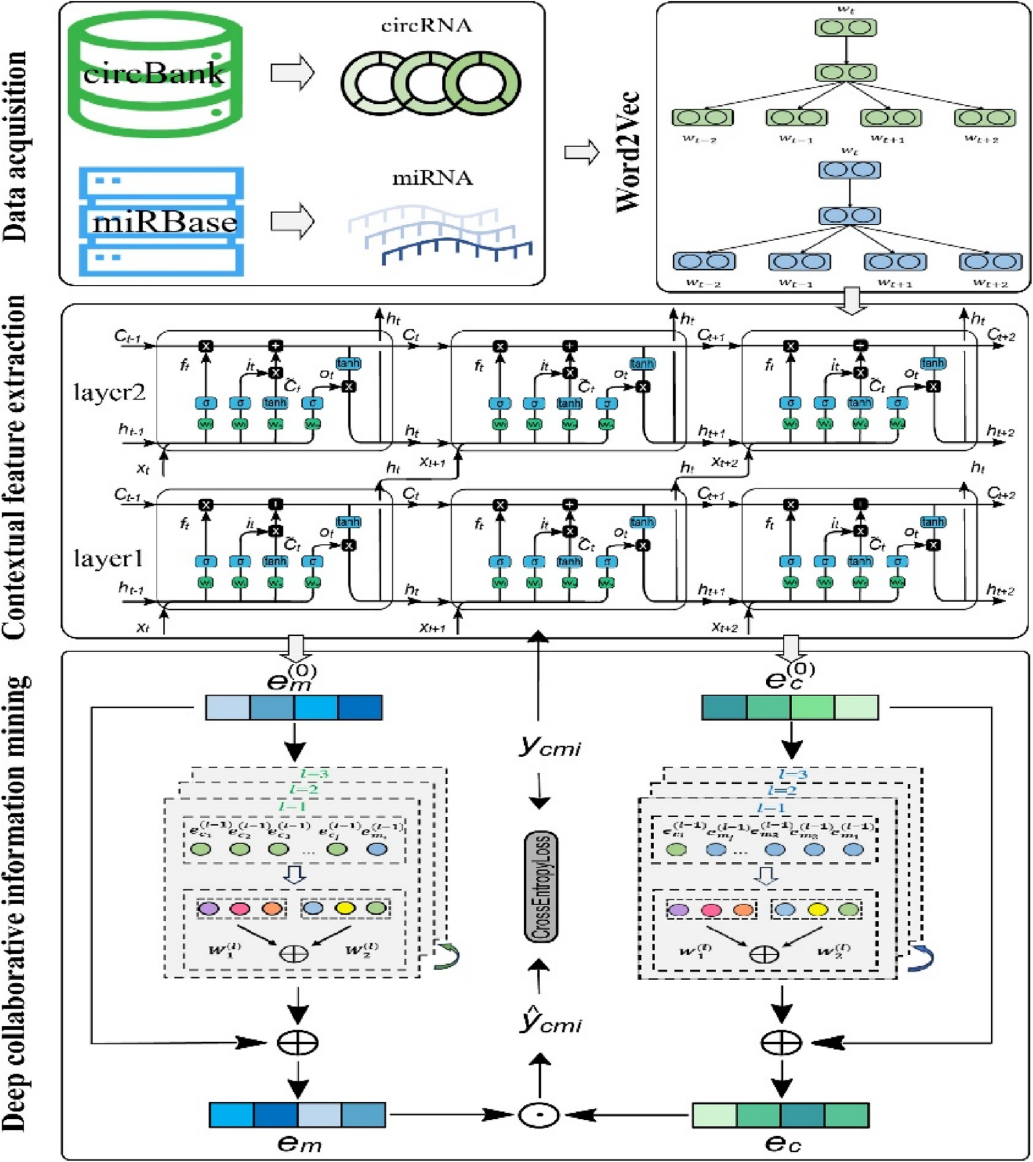
DGCLCMI model overall architecture diagram

Experimental results on three well-established datasets demonstrate that our DGCLCMI algorithm achieves outstanding performance compared to previous methods. Additionally, the ablation study and case analysis of the two main improvements proposed in this paper validate the effectiveness of our algorithm. Therefore, DGCLCMI is an innovative and high-performance circRNA-miRNA association prediction tool (the model architecture diagram is shown in Fig. 1), which is expected to advance research in this field. The contributions of this paper are summarized as follows:

1. For the first time, the neural collaborative filtering algorithm is introduced and improved to mine deep interaction features of circRNA-miRNA.
2. An innovative joint optimization framework for deep collaborative feature mining and underlying feature extraction is constructed.
3. Experiments on three well-recognized datasets show that DGCLCMI achieves superior performance compared to existing methods.

## Results and discussion

### Performance of the proposed algorithm

**Fig. 2:**
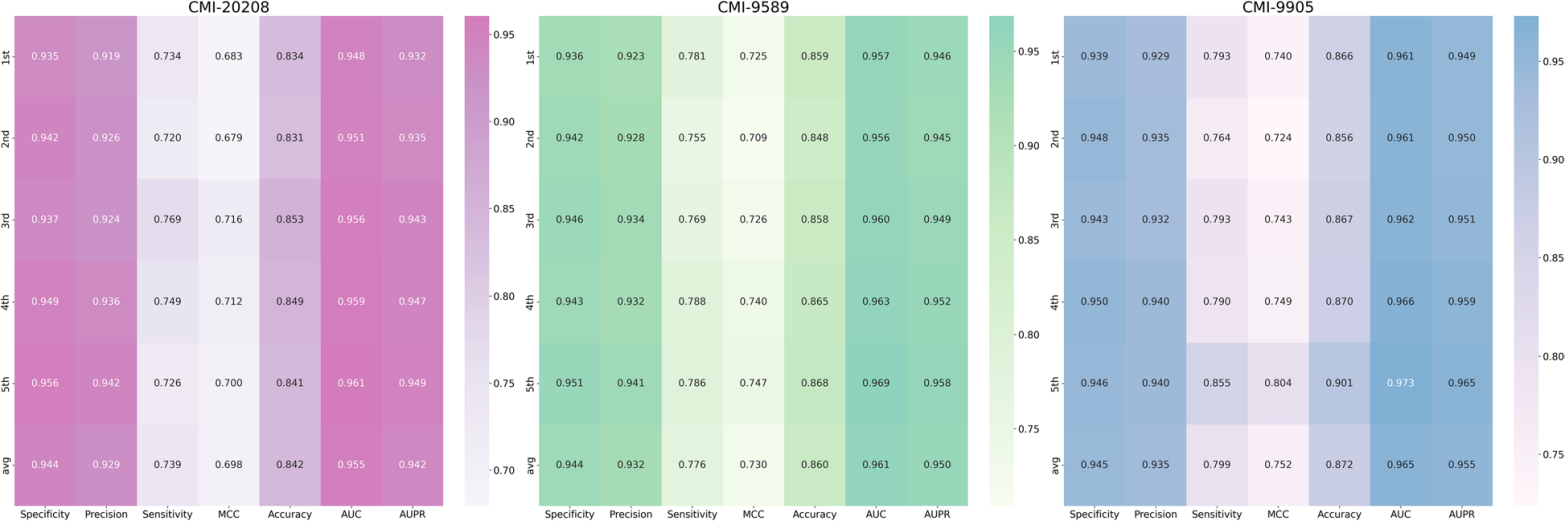
Heatmap showing fivefold cross-validation results of our algorithm across three datasets

**Fig. 3:**
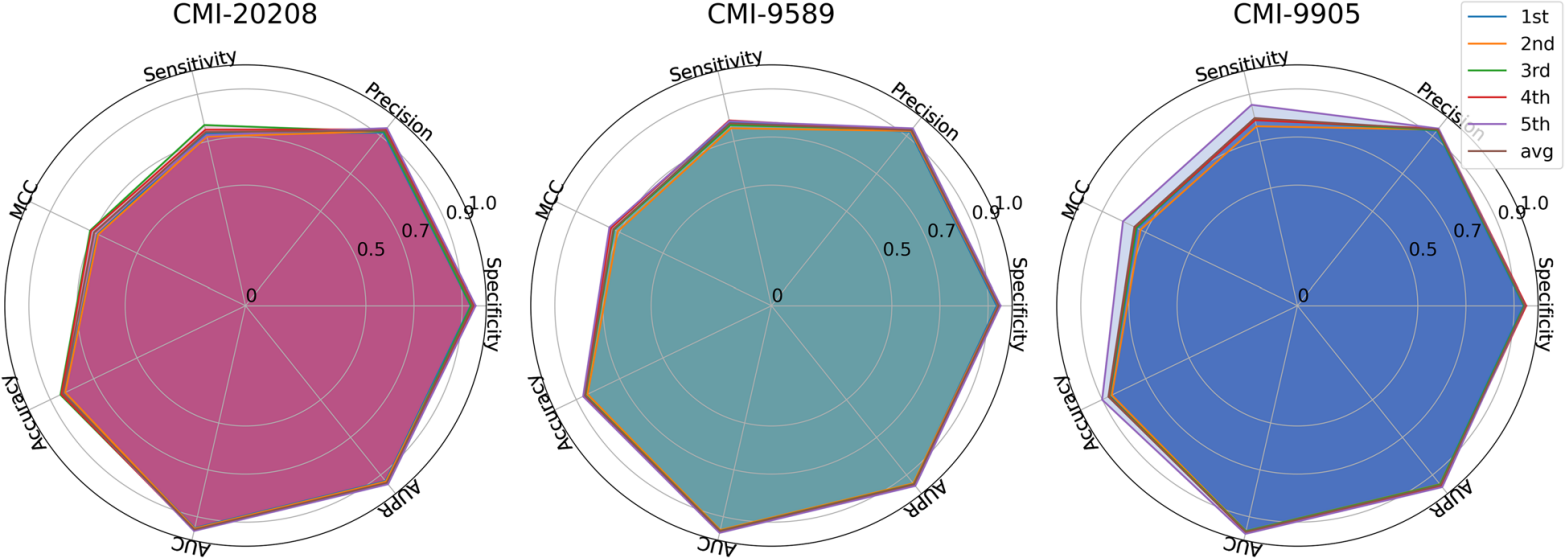
Radar chart showing fivefold cross-validation results of our algorithm across three datasets

We summarized publicly available datasets commonly used in previous research, including CMI-20208, CMI-9589, and CMI-9905, and conducted fivefold cross-validation on these datasets using the proposed algorithm. The obtained performance is measured using seven indicators from various aspects, and the results are shown in Fig. 2 and Table 1. At the same time, for the convenience of intuitive evaluation, we have also visualized the results, as shown in Fig. 3.

**Table 1:** Average performance of our algorithm on the CMI-20208, CMI-9589, and CMI-9905 datasets

Dataset	Specificity	Precision	Sensitivity	MCC	Accuracy	AUC	AUPR
CMI-20208	0.9439	0.9294	0.7394	0.6981	0.8417	0.9546	0.9415
CMI-9589	0.9437	0.9318	0.7758	0.7297	0.8596	0.9610	0.9501
CMI-9905	0.9452	0.9352	0.7988	0.7521	0.8719	0.9645	0.9546
Average	0.9443	0.9321	0.7713	0.7266	0.8577	0.9600	0.9487

It can be observed that our algorithm achieved good performance in the fivefold cross-validation of both the 9589 and 20,208 CMI correlated pairs, demonstrating excellent performance across all metrics. Notably, the algorithm showed particularly strong performance in the specificity and precision metrics. As is well-known, higher specificity indicates a stronger ability of the model to recognize negative samples, leading to a lower misdiagnosis rate, while higher precision reflects better accuracy in identifying positive cases. Additionally, the algorithm demonstrates reasonably good performance in the sensitivity metric, which measures the recall of positive samples. Consequently, the algorithm achieves outstanding overall performance in the comprehensive AUC metric, with fivefold average AUCs of 0.9546, 0.9610, and 0.9645 for the CMI-20208, CMI-9589, and CMI-9905 datasets, respectively, yielding an average AUC of 0.9600. A high AUC value suggests that the model can effectively differentiate between positive and negative samples across different thresholds.

As is known, a higher specificity value indicates a stronger ability of the model to identify negative samples, resulting in a lower misdiagnosis rate; a higher precision value reflects the model’s accuracy in identifying positive cases. Additionally, the algorithm displayed satisfactory performance in the sensitivity metric, which measures the recall of positive samples. Therefore, the algorithm achieved excellent overall performance in the AUC (area under che Curve) metric, with the fivefold average AUC on the CMI-20208, CMI-9589, and CMI-9905 datasets being 0.9543, 0.9611, and 0.9647, respectively, with an average of 0.9600. A high AUC value indicates that the model was able to effectively distinguish between positive and negative samples at different thresholds.

Of course, compared to other metrics, the MCC result may not stand out as much. As a metric that considers true positives, false positives, false negatives, and true negatives, MCC imposes higher demands on the algorithm’s performance. Nevertheless, our algorithm demonstrates significant improvement in this metric compared to state-of-the-art methods (related comparison experiments are presented in the next section). In summary, the proposed algorithm exhibits strong performance in the CMI prediction task and offers valuable insights for the exploration of potential CMIs.

### Performance comparison with existing prediction algorithms

**Fig. 4:**
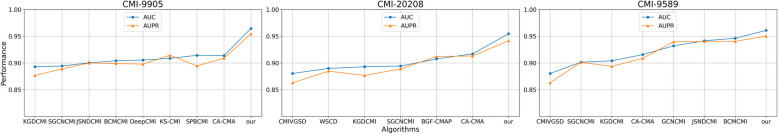
AUC and ACPR curve comparison of our algorithm with existing prediction algorithms on three datasets

In this section, we compare the proposed method with several advanced CMI prediction models using AUC and AUPR indicators on three datasets, as shown in Fig. 4. Our method outperforms previous algorithms in terms of AUC across all datasets, with significant improvements on certain datasets. Specifically, on the CMI-9905, CMI-20208, and CMI-9589 datasets, it surpasses the second-best method by 5.07%, 3.76%, and 1.47% in AUC, and 4.58%, 2.84%, and 0.96% in AUPR, respectively.

We observed that most of the previous algorithms, such as BGF-CMAP, SPBCMI, KS-CMI, DeepCMI, BCMCMI, and JSNDCMI, use the gradient boosting tree (GBT) algorithm or its variants as classifiers for the captured features. Although these classifiers iteratively reduce prediction errors by constructing multiple decision trees and making final predictions using weighted averages, they generally only handle “static” features. These methods cannot dynamically generate new features or adjust them in real time, limiting their ability to extract deep dynamic features. For time-series or dynamic features, corresponding feature creation must occur during the data preprocessing stage. This limitation hinders the algorithm’s capacity to extract the most representative features that would maximize performance, ultimately affecting the prediction results. In contrast, our model utilizes a neural graph collaborative filtering framework combined with a bottom-layer feature extraction network. This collaborative approach captures deep information in circRNA-miRNA interactions, which is then stored in their respective embeddings. As a result, this dynamic training mechanism enables comprehensive exploration of association patterns in CMI, leading to significant performance improvements over static methods (related ablation experiments and evaluations of different classifiers are discussed in the next section).

We also found that algorithms using autoencoders or their variants, such as SGCNCMI, KGDCMI, CMIVGSD, and CA-CMA, which perform further feature extraction, generally fail to achieve optimal performance. While autoencoders can automatically extract low-dimensional feature representations from high-dimensional data without labels, the features they generate often lack clear practical or physical significance. Moreover, since there are no explicit supervisory signals, the extracted features are difficult to directly apply to downstream tasks. To obtain useful feature embeddings for specific tasks, significant manual intervention (e.g., tuning, adding constraints) is usually required. In contrast, the joint optimization and adaptive feature-capturing network proposed in this paper offer notable practical advantages for task-oriented feature extraction.

For a more comprehensive comparison, we present additional metrics on the CMI-9905 and CMI-20208 datasets, as shown in Tables 2 and 3. It is clear that, compared to existing models, our algorithm exhibits superior performance across all metrics. In CMI-9905, compared to the CA-CMA model proposed in 2024, our method achieves approximately a 12% improvement in Specificity, a 10% improvement in Precision, and a 6% increase in overall MCC. In the larger CMI interaction network (CMI-20208), compared to the BGF-CMAP model proposed in 2024, our algorithm still achieves about an 11% improvement in Specificity, a 10% improvement in Precision, and about a 4% increase in overall MCC. These results further highlight the robustness of our algorithm, demonstrating the effectiveness of the deep graph collaboration method proposed in this paper.

**Table 2:** Comparison of four performance metrics between the proposed and existing algorithms on CMI-9905

Algorithm	Accuracy	Precision	Specificity	MCC
KGDCMI	0.8265	0.8435	0.8510	0.6538
JSNDCMI	0.8231	0.8232	0.8249	-
DeepCMI	0.8244	0.8290	-	0.6496
BCMCMI	0.8316	0.8083	-	0.6670
KS-CMI	0.8343	0.8366	-	-
SPBMCI	0.8405	-	-	0.6864
CA-CMA	0.8399	0.8328	0.8289	0.6804
Our	**0.8719**	**0.9352**	**0.9452**	**0.7521**

**Table 3:** Comparison of four performance metrics between the proposed and existing algorithms on CMI-20208

Algorithm	Accuracy	Specificity	Precision	MCC
WSCD	0.8161	0.8132	0.8143	0.6323
BGF-CMAP	0.8290	0.8384	0.8353	0.6581
Our	**0.8417**	**0.9439**	**0.9294**	**0.6981**

### Ablation experiments and analysis

#### Performance evaluation of different classifier algorithms

**Fig. 5:**
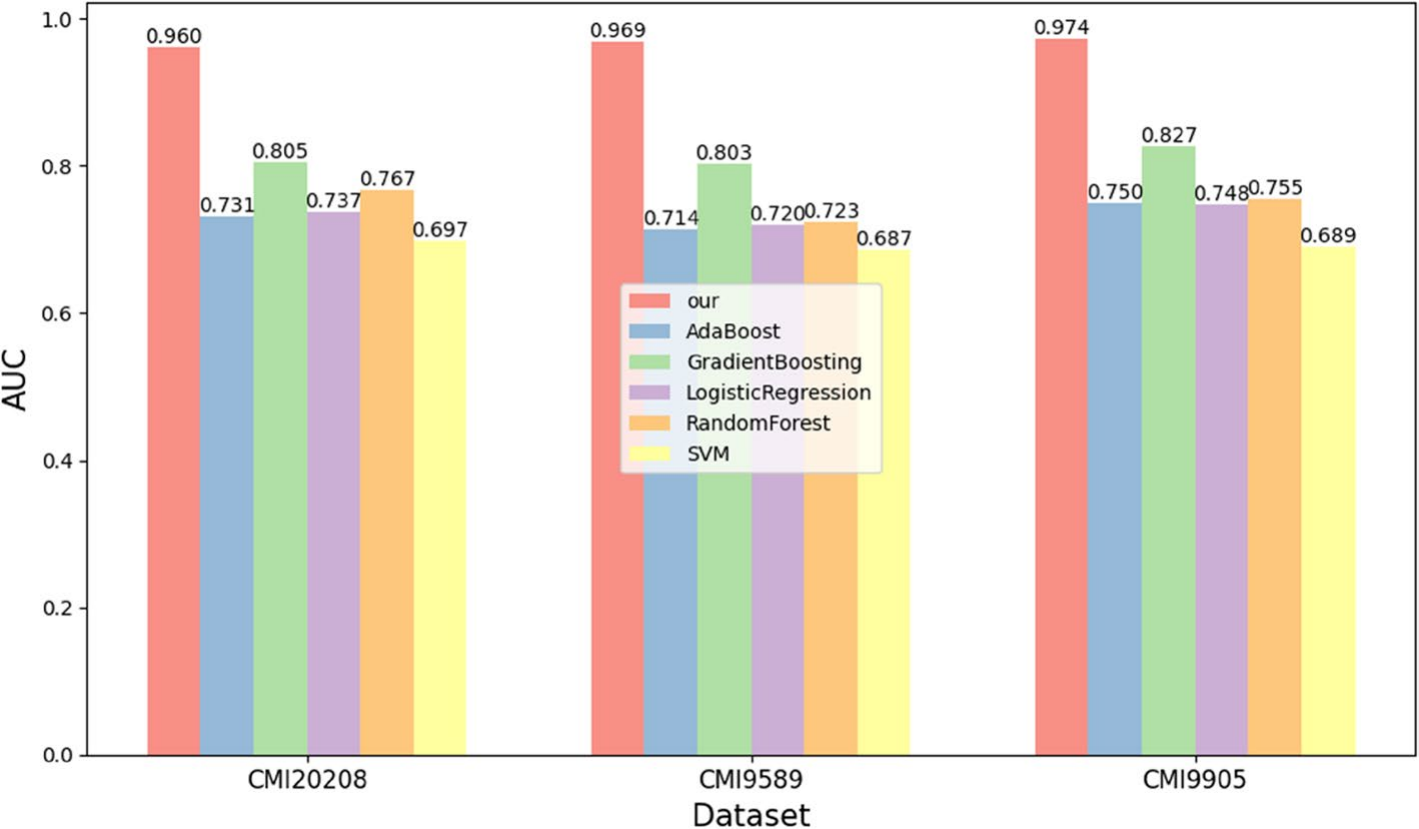
Performance comparison of DGCLCMI and other classifiers on the same features numerically processed

To highlight the superiority of the proposed algorithm, which introduces and improves the neural collaborative filtering algorithm to mine deep interaction features of CMI, we perform an ablation analysis comparing it with commonly used classifier algorithms in previous studies such as AdaBoost, Gradient Boosting, Logistic Regression, Random Forest, and SVM. This comparison is based on the same numerically processed sequence features, as shown in Fig. 5. The results show that our dynamic and unified model performs exceptionally well across all datasets, standing out clearly. This further validates the earlier point that our algorithm surpasses “static” classifiers, which are based on decision tree ensemble learning algorithms and cannot dynamically adjust features during training.

AdaBoost improves performance by adjusting sample weights, while Random Forest uses Bagging (Bootstrap Aggregating) to train multiple decision trees in parallel, with the final result determined by the weighted sum of all trees. Gradient Boosting optimizes the model by gradually reducing residuals. However, these classifiers can only perform classification based on existing features and cannot adapt features based on label information, leaving room for performance improvement. Logistic Regression, being a linear model, is suitable for linearly separable data, but for the highly nonlinear CMI prediction task, it struggles to capture such complexity effectively.

We also observed that SVM did not perform well in this experiment. This might be because SVM typically excels in small sample sizes and high-dimensional, linearly separable scenarios. However, in large-scale datasets with more noise, it may not be as efficient as other algorithms, such as Random Forest or neural networks.

#### Performance evaluation of different feature extraction algorithms

**Fig. 6:**
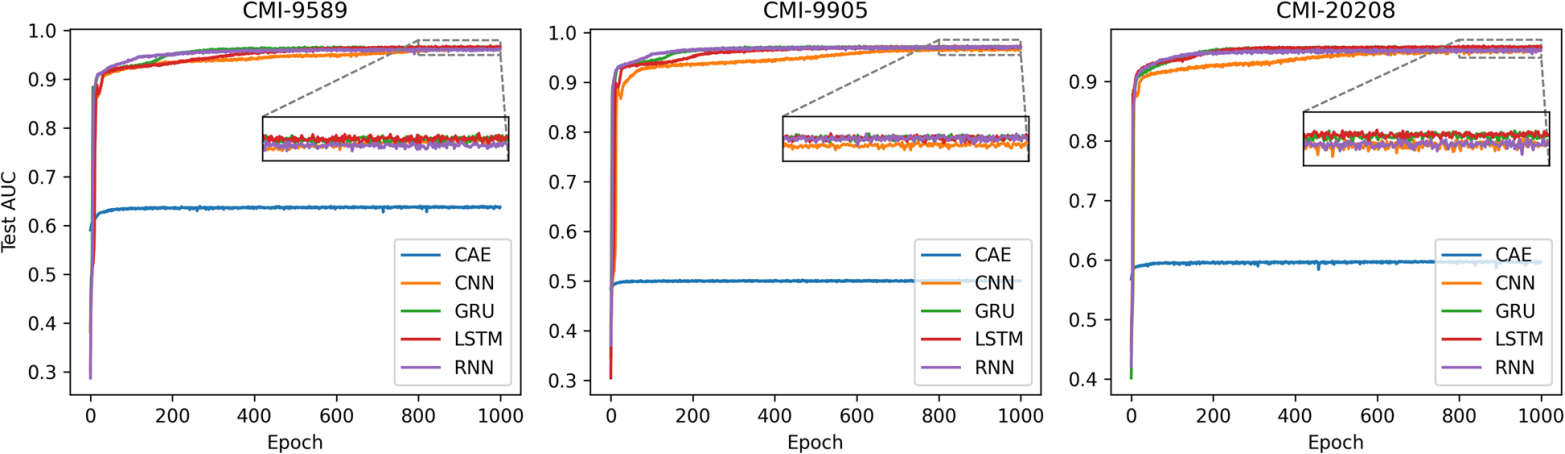
Performance evaluation of various feature extraction algorithms within the same deep collaborative information mining framework

In this section, to validate the contribution of the deep collaborative feature mining and bottom-layer feature extraction joint optimization framework proposed in this paper, as well as the chosen feature extraction algorithm LSTM, we compare and perform an ablation analysis on various feature extraction algorithms used in previous studies, such as CNN, RNN, GRU, and CAE, using the proposed neural graph collaborative filtering model (Fig. 6). We observe that the feature extraction algorithms paired with the neural graph collaborative filtering model (except for CAE) all achieved excellent performance under the joint optimization framework. However, the performance of the non-jointly optimized CAE is underwhelming, likely because the low-dimensional features captured by the autoencoder lack clear meaning and do not have direct constraints for optimization based on the final task, resulting in suboptimal performance.

Although CNN, RNN, LSTM, and GRU show very similar performance across the three datasets, the red curve stands out slightly, achieving the best performance. The reason may be that, in time-series data, CNN extracts patterns within local time windows using one-dimensional convolutional kernels, similar to how local spatial features are extracted in images. It can capture the local correlations between different time steps in the sequence. However, for long-term dependency problems, it typically requires increasing the number of convolution layers or using a larger receptive field (filter size), but this does not fully address the long-range dependency issue. As a result, CNN’s performance ranks lower among the four algorithms. The remaining three algorithms—RNN, LSTM, and GRU—are commonly used for sequence data processing. RNN is the most basic recurrent neural network structure, processing data step by step in a sequence by sharing the same weights. Each time step receives the current input and the hidden state from the previous time step. However, RNN suffers from gradient vanishing or explosion problems when processing long sequences, making it difficult to capture long-range dependencies. LSTM was specifically designed to address the long-range dependency issue in standard RNNs. It introduces a “cell state” to directly pass information and uses a “gate mechanism” to control the flow of information. LSTM effectively remembers long-term information and selectively “forgets” irrelevant data, making it suitable for long-sequence tasks. However, due to its complex gating structure, LSTM has a higher computational cost and longer training time (as shown in the figure, LSTM is the slowest to converge among the three). GRU is a simplified version of LSTM, merging some of the gating mechanisms in LSTM to reduce the number of parameters.

Although the performance difference between GRU and LSTM is not significant in the experiments of this paper, the simpler structure of GRU may result in slightly weaker performance when handling long-range dependencies in very long sequences. Therefore, the model in this paper selects LSTM, in combination with the neural graph collaborative filtering model, as the primary model for capturing deep graph collaborative information.

### Case study

In order to further validate the proposed algorithm, which aids in the exploration of unknown associations, we conduct a case study in this section. First, we train the algorithm on CMI data with known labels and then predict whether there is an interaction between unknown circRNA and miRNA pairs. Unknown samples with high confidence were selected and further tested by consulting relevant literature and the CircInteractome database. If relevant literature or database data existed, the sample was labeled as “Confirmed”; otherwise, it was labeled as “Unconfirmed”. The results are shown in Table 4. It can be seen that, of the 20 unknown correlated sample pairs in the table, 18 have been confirmed, and the remaining 2 may also be confirmed in the future through practical testing. Overall, the proposed algorithm is able to provide potential interaction pairs with high confidence, effectively narrowing down the scope of candidates and reducing the cost of experimental trial and error, demonstrating a strong ability to identify potential CMIs.

**Table 4:** Twenty high-confidence CMIs predicted by DGCLCMI

No	circRNA	miRNA	Evidence
1	hsa_circ_0009566	hsa-miR-646	Confirmed
2	hsa_circ_0010085	hsa-miR-658	Confirmed
3	hsa_circ_0023020	hsa-miR-326	Confirmed
4	hsa_circ_0024899	hsa-miR-663b	Confirmed
5	hsa_circ_0030874	hsa-miR-658	Confirmed
6	hsa_circ_0041605	hsa-miR-361-3p	Confirmed
7	hsa_circ_0049497	hsa-miR-1183	Confirmed
8	hsa_circ_0049497	hsa-miR-3182	Unconfirmed
9	hsa_circ_0050270	hsa-miR-1289	Confirmed
10	hsa_circ_0050501	hsa-miR-658	Confirmed
11	hsa_circ_0056345	hsa-miR-1299	Confirmed
12	hsa_circ_0063366	hsa-miR-1224-3p	Confirmed
13	hsa_circ_0063366	hsa-miR-6504-5p	Unconfirmed
14	hsa_circ_0069972	hsa-miR-326	Confirmed
15	hsa_circ_0073951	hsa-miR-450b-3p	Confirmed
16	hsa_circ_0075975	hsa-miR-361-3p	Confirmed
17	hsa_circ_0078807	hsa-miR-1183	Confirmed
18	hsa_circ_0079478	hsa-miR-1224-3p	Confirmed
19	hsa_circ_0081151	hsa-miR-587	Confirmed
20	hsa_circ_0081156	hsa-miR-326	Confirmed

## Conclusions

Circular RNAs (circRNAs) facilitate the expression of specific target genes by modulating miRNA activity and alleviating miRNA-mediated suppression, thereby influencing critical cellular processes including proliferation, differentiation, and apoptosis. Therefore, studying circRNA-miRNA interactions is crucial for deciphering intracellular regulatory networks and understanding complex gene expression mechanisms. Although existing computational models have been proposed for predicting these interactions, they predominantly suffer from two fundamental limitations:

They overlook the extraction of interaction collaboration features and fail to train the feature extraction network based on this information, which affects the performance of the algorithms. To resolve these drawbacks, we introduce DGCLCMI, a new deep graph collaborative learning framework. Specifically, we enhanced the NGCF model to capture deep collaborative features of CMIs and employed these signals to guide the extraction of representative features from biological sequences for prediction. In comprehensive evaluations across three benchmark CMI datasets, our algorithm demonstrates superior performance over state-of-the-art methods. In conclusion, our framework exhibits strong CMI prediction performance, which facilitates the exploration of unknown CMIs, thereby revealing the underlying disease-regulating networks and advancing the development of early diagnosis and targeted therapies.

## Methods

### Datasets

In this study, to better evaluate the performance of the proposed algorithm and facilitate comparison with existing prediction models, we use the publicly available, experimentally validated datasets CMI-9905, CMI-9589, and CMI-20208, which have been employed in previous studies [63]. This allows for a performance evaluation under the same benchmark datasets. The detailed data are presented in Table 5. These datasets were sourced from the circBank [66] (http://www.circbank.cn/) and miRBase [67] (https://www.mirbase.org/) databases, containing experimentally verified circRNA-miRNA interaction data. For model training and evaluation, we also randomly selected an appropriate number of negative samples to construct the circRNA-miRNA association matrix D and performed a fivefold cross-validation to randomly split the dataset into training and testing sets.

**Table 5:** Datasets details

Dataset	CMI-9589	CMI-9905	CMI-20208
CircRNA	2115	2346	3569
MiRNA	821	962	1152
CMI	9589	9905	20,208

### Proposed model architecture

The architecture diagram of our prediction model is shown in Fig. 1, which primarily consists of four main components: preliminary sequence feature extraction, sequence time dependence capturing, circRNA-miRNA deep collaboration information mining, and CMI interaction predicting. In the following sections, we will describe each of these four modules in detail to more clearly illustrate the internal structure and functionality of the algorithm proposed in this paper.

#### Sequence numerical processing

The original circRNA and miRNA sequences are only composed of four bases: A, G, C, and U, making the data highly specialized and difficult to interpret. To convert these sequences into numerical features that can be understood by machines and facilitate further analysis, we adopt the Skip-gram model, which is commonly used for word representation in natural language processing (NLP), for the initial extraction of sequence numerical features.

Skip-gram [65], as a training form of Word2Vec, uses the center word ${w}_{t}$ of a given sentence to maximize the corresponding context word ${w}_{t+j}$ of the predicted position to train the word representation (the formula is expressed as follows). Skip-gram is used in many NLP tasks and has achieved significant performance improvement.

1 $$\frac{1}{T}\sum\limits_{t = 1}^{T} {\sum\limits_{ - k \le j \le k,j \ne 0} {\log } } P\left( {w_{t + j} w_{t} } \right)$$

For the encoding of circRNA and miRNA, consistent with CA-CMA, we treated each base as a word, set the dimension of the word vector to 64, and used the Skip-gram model to train the embedding feature representations for the four bases of the corresponding RNA sequences.

#### Sequence contextual feature extraction

In order to capture the temporal contextual features in the sequence, we employ the well-known LSTM network in the field of NLP to regulate information flow through a gating mechanism. This mechanism filters valuable features, removes noise data, and effectively retains long-range dependencies. The key components of LSTM include the forget gate, input gate, and output gate, which determine how information is retained or discarded at each time step.

Specifically, the forget gate controls which information should be discarded from the cell state, and its computation is as follows:

2 $$f_{t} = \sigma \left( {W_{f} \cdot \left[ {h_{t - 1} ,x_{t} } \right] + b_{f} } \right)$$

where ${f}_{t}$ is the output of the forget gate. $\sigma$ is the sigmoid activation function. ${W}_{f}$ is the weight matrix of the forget gate. ${h}_{t-1}$ is the hidden state of the previous time step. ${x}_{t}$ is the current input. ${b}_{f}$ is the bias term.

The input gate determines which new information should be added to the cell state. This process consists of two steps: (1) generating new candidate information; (2) updating the cell state based on the input gate. First, the candidate cell states are calculated:

3 $$\tilde{C}_{t} = \tanh \left( {W_{C} \cdot \left[ {h_{t - 1} ,x_{t} } \right] + b_{C} } \right)$$

where ${\widetilde{C}}_{t}$ represents the new candidate information, and tanh is the hyperbolic tangent function.

Input gate calculation:

4 $$i_{t} = \sigma \left( {W_{i} \cdot \left[ {h_{t - 1} ,x_{t} } \right] + b_{i} } \right)$$

Updated cell status:

5 $$C_{t} = f_{t} \cdot C_{t - 1} + i_{t} \cdot \tilde{C}_{t}$$

Output gate calculation:

6 $$o_{t} = \sigma \left( {W_{o} \cdot \left[ {h_{t - 1} ,x_{t} } \right] + b_{o} } \right)$$

Updated hidden status:

7 $$h_{t} = o_{t} \cdot \tanh \left( {C_{t} } \right)$$

To effectively capture the semantic features of circRNA and miRNA sequences, we set the input feature dimension of the LSTM network to 64 and the hidden state dimension to 256. Additionally, we stack two layers of LSTM networks to enhance the model’s capability to capture dependencies. The final output is compressed and refined using a fully connected layer, yielding 128-dimensional feature representations.

#### circRNA-miRNA deep collaborative information mining

Inspired by the NGCF model proposed by Wang et al. [68] and the MLNGCF model [69], we apply the NGCF model with improvements to extend its applicability to CMI prediction tasks. Moreover, in previous studies employing NGCF, feature extraction, and collaborative information mining were treated as independent modules and trained separately. For example, the MLNGCF model first applied a deep autoencoder network (DAE) to extract low-dimensional embeddings from circRNA-circRNA and disease-disease similarity graphs. These embeddings were then fed into NGCF to mine collaborative information. However, the feature extraction and collaborative information mining modules were trained separately, preventing NGCF from leveraging the mined collaborative information to refine feature embeddings, thereby limiting their representativeness.

In this study, we integrate the feature extraction module and the deep interactive information mining module into a unified optimization framework. By capturing the sequence context in the previous section, we obtained initial representative features of circRNA and miRNA sequences. We then propose a deep collaboration model that further explores circRNA-miRNA interactions to refine sequence embeddings based on CMI interaction data. Additionally, the mined collaboration information is leveraged to guide the extraction of sequence context features through gradient backpropagation. Specifically, we employ GNN to construct a multi-layer message passing mechanism, capturing circRNA-miRNA collaborative signals based on the CMI graph structure and optimizing the learned embeddings of circRNA and miRNA.

#### Construction of multi-layer message propagation mechanism

Drawing inspiration from recommendation systems, we generalize a similar concept by treating miRNAs interacting with circRNA as features of circRNA, thereby measuring the similarity between different miRNAs. Additionally, the interaction between circRNA and miRNA reflects the binding preference of circRNA for specific miRNAs.

#### Message transfer

To achieve this, we design a message passing mechanism for information exchange between circRNA and miRNA. Given a set of CMI data (c,m) and their corresponding embeddings ${e}_{c}$ and ${e}_{m}$, the message propagation mechanism encoding m → c integrates the embedding information of ${e}_{m}$ and interactive encoding between ${e}_{c}$ and ${e}_{m}$, formulated as follows:

8 $$m_{m \to c} = f\left( {e_{m} ,e_{c} ,p_{mc} } \right)$$

9 $$m_{m \to c} = \frac{1}{{\sqrt {\left| {{\mathcal{N}}_{c} } \right|\left| {{\mathcal{N}}_{m} } \right|} }}\left( {W_{1} e_{m} + W_{2} \left( {e_{m} \odot e_{c} } \right)} \right)$$

where ${p}_{mc}$ represents a loss factor in the message passing process, ${N}_{c}$ and ${N}_{m}$ denote the respective first-order neighborhoods, and ${W}_{1}$ and ${W}_{2}$ are the weight matrices for information propagation, responsible for extracting useful information from the corresponding elements.

#### Message aggregation

According to the miRNA message transmission ${m}_{m\to c}$, the original embedded information ${e}_{c}$ is aggregated to obtain a new representation ${e}_{c}^{(1)}$. The weight matrix ${W}_{1}$ remains consistent with the one used in previous layers.

10 $${\mathbf{e}}_{c}^{(1)} = {\text{LeakyReLU}} \left( {W_{1} e_{c} + \sum\limits_{{i \in {\mathcal{N}}_{c} }} { m_{m \to c} } } \right)$$

By stacking the aforementioned $l$ message propagation layers, circRNA and miRNA can receive collaborative signals propagated from their $l$-order neighbors, thereby capturing higher-order interaction information of CMI. Moreover, the model explicitly encodes deep collaborative information into the sequence representations, which is crucial for the subsequent evaluation of the association strength between circRNA and miRNA.

11 $$\left\{ \begin{gathered} m_{m \to c}^{(l)} = \frac{1}{{\sqrt {\left| {{\mathcal{N}}_{c} } \right|\left| {{\mathcal{N}}_{m} } \right|} }}\left( {W_{1}^{(l)} e_{m}^{{(l{ - }1)}} + W_{2}^{(l)} \left( {e_{m}^{{(l{ - }1)}} \odot e_{c}^{{(l{ - }1)}} } \right)} \right) \hfill \\ {\mathbf{e}}_{c}^{(l)} = {\text{LeakyReLU}} \left( {W_{1}^{(l)} e_{c}^{{(l{ - }1)}} + \sum\limits_{{i \in {\mathcal{N}}_{c} }} { m_{m \to c}^{(l)} } } \right) \hfill \\ \end{gathered} \right.$$

To facilitate parallelization, we employ a matrix representation for layer-wise message propagation based on the GNN computational framework. Specifically, the interaction matrix R is derived from the training set of CMI data, and we construct the adjacency matrix $A\in {R}^{\left(n+m\right)\times \left(n+m\right)}$ of the collaboration graph, where $n$ and $m$ denote the numbers of miRNAs and circRNAs, respectively. The Laplacian matrix is then computed and normalized. Based on the computational framework of GNN, the matrix formulation for message propagation can be derived accordingly.

12 $${\mathbf{A}}_{cm} = \left[ {\begin{array}{*{20}c} 0 & {\text{R}} \\ {{\mathbf{R}}^{{ \top }} } & 0 \\ \end{array} } \right]$$

13 $${\mathcal{L}} = {\mathbf{D}}^{{ - \frac{1}{2}}} \left( {{\text{A}}_{cm} + {\text{I}}} \right){\text{D}}^{{ - \frac{1}{2}}}$$

14 $${\mathbf{E}}^{(0)} = [e_{{m_{1} }} ,...,e_{{m_{n} }} ,e_{{c_{1} }} ,...,e_{{c_{m} }} ] \in R^{(n + m) \times d}$$

15 $${\mathbf{E}}^{(l)} = {\text{LeakyReLU}} \left( {{\mathcal{L}}{\mathbf{E}}^{(l - 1)} {\mathbf{W}}_{1}^{(l)} + \left( {{\mathbf{E}}^{(l - 1)} \odot {\mathcal{L}}{\mathbf{E}}^{(l - 1)} } \right){\mathbf{W}}_{2}^{(l)} } \right)$$

where $I$ denotes the identity matrix and D represents the degree matrix. Through the $l$-layer message passing process, we can obtain high-order circRNA-miRNA collaborative signals and encode them into the corresponding sequence embeddings as representative sequence features.

#### Prediction of CMI interaction

After sequence semantic feature extraction and deep interaction information mining, we can obtain the representative features of circRNA and miRNA sequences in a shared feature space. Thus, to evaluate the interaction between circRNAs and miRNAs, we directly calculate the inner product of their corresponding feature embeddings, ${e}_{c}$ and ${e}_{m}$, from which interaction scores can be derived. To closely fit the training data and capture the underlying CMI mechanism, we use cross-entropy loss as a measure of the difference between the model's predicted and true values and apply gradient backpropagation to update the model parameters.

16 $$\hat{y}_{{{\text{CMI}}}} = e_{m}^{{\text{T}}} e_{c}$$

17 $$loss = \frac{1}{k}\sum\limits_{i}^{k} {\left[ {y_{i} \log \hat{y}_{{{\text{CMI}}}} + \left( {1 - y_{i} } \right)\log \left( {1 - \hat{y}_{{{\text{CMI}}}} } \right)} \right]}$$

### Experimental setup and evaluation metrics

The experimental results of the model proposed in this paper are based on the following settings: the learning rate $lr$ is set to 1e − 4, the batch_size is 128, and the graph collaboration network employs three message-passing layers, each with a size of 64. The node_dropout and mess_dropout are both set to 0.1. The Adam optimizer is used to train the entire model with betas = (0.9, 0.999), eps = 1e − 8. In addition, the performance of the algorithm is evaluated using a variety of metrics, including precision (Prec.), specificity (Spec.), sensitivity (Sens.), accuracy (Accu), Matthews correlation coefficient (MCC), area under the curve (AUC), and area under the precision-recall curve (AUPR), providing a comprehensive evaluation of the model’s performance.

## Data Availability

All data generated or analysed during this study are included in this published article, its supplementary information files and publicly available repositories: Zenodo [70] (https://zenodo.org/records/15063000) and GitHub (https://github.com/cc646201081/DGCLCMI).

## Abbreviations

circRNA: Circular RNA
miRNA: MicroRNA
CMI: CircRNA-miRNA interaction
CNN: Convolutional neural network
RNN: Recurrent neural network
GNN: Graph neural network
LSTM: Long short-term memory
GRU: Gated recurrent unit
GIP: Gaussian interaction profile
SVD: Singular value decomposition
VGAE: Variational graph autoencoder
CBOW: Continuous bag of words
SDNE: Structural deep network embedding
DNN: Deep neural network
GCN: Graph convolutional networks
SAE: Sparse autoencoder
DAE: Denoising autoencoders
GBDT: Gradient boosting decision tree
BERT: Bidirectional Encoder Representations from Transformers
CatBoost: Categorical boosting
XGBoost: Extreme gradient boosting
CAE: Convolutional autoencoders
GF: Graph factorization
LINE: Large-scale information network embedding
NGCF: Neural graph collaborative filtering model
GBT: Gradient boosting tree
AUC: Area under the curve
MCC: Matthews correlation coefficient
AUPR: Area under the precision-recall curve
NLP: Natural language processing
